# A dCas9-Based System Identifies a Central Role for Ctf19 in Kinetochore-Derived Suppression of Meiotic Recombination

**DOI:** 10.1534/genetics.120.303384

**Published:** 2020-08-25

**Authors:** Lisa-Marie Kuhl, Vasso Makrantoni, Sarah Recknagel, Animish N. Vaze, Adele L. Marston, Gerben Vader

**Affiliations:** * Department of Mechanistic Cell Biology, Max Planck Institute of Molecular Physiology, Dortmund 44227, Germany; † The Wellcome Centre for Cell Biology, Institute of Cell Biology, School of Biological Sciences, University of Edinburgh, EH9 3BF, United Kingdom; ‡ International Max Planck Research School (IMPRS) in Chemical and Molecular Biology, Max Planck Institute of Molecular Physiology, Dortmund 44227, Germany

**Keywords:** CRISPR, dCas9, kinetochore, meiosis, recombination

## Abstract

In meiosis, crossover (CO) formation between homologous chromosomes is essential for faithful segregation. However, misplaced meiotic recombination can have catastrophic consequences on genome stability. Within pericentromeres, COs are associated with meiotic chromosome missegregation. In organisms ranging from yeast to humans, pericentromeric COs are repressed. We previously identified a role for the kinetochore-associated Ctf19 complex (Ctf19c) in pericentromeric CO suppression. Here, we develop a dCas9/CRISPR-based system that allows ectopic targeting of Ctf19c-subunits. Using this approach, we query sufficiency in meiotic CO suppression, and identify Ctf19 as a mediator of kinetochore-associated CO control. The effect of Ctf19 is encoded in its NH2-terminal tail, and depends on residues important for the recruitment of the Scc2-Scc4 cohesin regulator. This work provides insight into kinetochore-derived control of meiotic recombination. We establish an experimental platform to investigate and manipulate meiotic CO control. This platform can easily be adapted in order to investigate other aspects of chromosome biology.

## Abstract (precis)

A dCas9-based system is developed to query the regulation of kinetochore-driven meiotic recombinational control....

FAITHFUL chromosome segregation in meiosis requires physical connections between initially unpaired homologous chromosomes (Petronczki *et al.* 2003). Such linkages are established through homologous recombination (HR)-mediated repair of programmed DNA double-strand breaks (DSBs) (Keeney 2001). Sequences that can act as HR repair templates for DSB lesions are found on the sister chromatid and the homologous chromosome, but only repair that uses the homologous chromosome as a template can result in exchange of flanking chromosomal arm regions of homologous chromosomes, yielding a crossover (CO). A CO, together with cohesin laid down distally to the recombination site, establishes the connection between homologs required for chromosome segregation in meiosis. The placement of crossovers is determined by the location of DSB activity and by repair decisions after DSB formation. Certain regions in the genome represent a risk to genome stability when faced with DSB repair or CO formation, and molecular systems are in place to control CO placement and thereby guard genomic stability during meiosis.

Centromeres are the regions of the chromosomes where kinetochores are nucleated. Kinetochores are multi-subunit chromatin-associated assemblies that coordinate microtubule-chromosome attachments, cell cycle control, and local chromosome organization (Musacchio and Desai 2017). DSB activity and CO formation in centromere-proximal regions (*i.e.*, pericentromeres) are suppressed in organisms ranging from yeast to humans (Nakaseko *et al.* 1986; Lambie and Roeder 1988; Centola and Carbon 1994; Mahtani and Willard 1998; Borde *et al.* 1999; Copenhaver *et al.* 1999; Puechberty *et al.* 1999; Gerton *et al.* 2000; Westphal and Reuter 2002; Blitzblau *et al.* 2007; Buhler *et al.* 2007; Gore *et al.* 2009; Saintenac *et al.* 2009; Ellermeier *et al.* 2010; Pan *et al.* 2011). Improper placement of COs in pericentromeres is associated with chromosome missegregation and aneuploidy (Koehler *et al.* 1996; Hassold and Hunt 2001; Lamb *et al.* 2005; Rockmill *et al.* 2006). The identity of pericentromeric sequences and chromatin diverges among different organisms. In many organisms, pericentromeres are made up of heterochromatin, and the establishment of this specialized chromatin is important for the suppression of meiotic DNA break formation and recombination (Ellermeier *et al.* 2010). We previously identified a functional contribution of budding yeast kinetochores to local suppression of CO formation in pericentromeric sequences (Vincenten *et al.* 2015). Within budding yeast kinetochores, the Ctf19c, the functional and molecular equivalent of the human constitutive centromere-associated network (CCAN) (Cheeseman and Desai 2008), plays a dual role in minimizing CO formation: Ctf19c (i) suppresses meiotic DSB formation surrounding kinetochores, and (ii) channels the repair of remaining DSBs into intersister-directed repair. These pathways lead to effective suppression of CO formation within pericentromeres (Vincenten *et al.* 2015; Kuhl and Vader 2019). Our experiments identified a role for pericentromeric cohesin-complexes (containing the meiosis-specific kleisin Rec8) in promoting intersister-mediated repair without affecting DSB activity (Vincenten *et al.* 2015). A recent study in fission yeast also identified a role for pericentromeric cohesin complexes in suppressing meiotic CO formation, although, in this case, the effect involved active suppression of local DSB activity (Nambiar and Smith 2018).

Kinetochores are cooperative assemblies of protein subcomplexes (Musacchio and Desai 2017). This characteristic can lead to pleiotropic loss of kinetochore subunits upon experimental interference with single components. For example, many Ctf19c subunits are codependent for their localization to the centromere (Pot *et al.* 2003; Pekgöz Altunkaya *et al.* 2016; Lang *et al.* 2018). This behavior has complicated delineating contributions of single kinetochore components to specific functional pathways, including the regulation of local CO suppression. To dissect contributions of kinetochore factors to the regulation of meiotic recombination, we developed a system that allows investigation of roles of kinetochore subunits in directing meiotic chromosome fragmentation and repair, by employing the dCas9/CRISPR system. Using this approach, we show that, in isolation, the Ctf19 subunit of the Ctf19c can mediate kinetochore-driven CO suppression. Previous work identified a key role for the unstructured NH_2_-terminal region of Ctf19 in mediating recruitment of the Scc2-Scc4 cohesin regulator (Hinshaw *et al.* 2015, 2017). Remarkably, this 30-amino-acid region of Ctf19 is sufficient to reduce CO formation at ectopic sites, suggesting a role for local regulation of cohesin function in influencing CO positioning.

## Materials and Methods

### Yeast strains and growth

All strains used were of the SK1 background and genotypes are given in Supplementary Data. Yeast cells were grown as described in Vincenten *et al.* (2015). Induction of synchronous meiosis was performed as described in Vader *et al.* (2011). Synchronous entry of cultures into the meiotic program was confirmed by flow cytometry-based DNA content analysis (see below). For expression of 3×Flag-dCas9 in meiosis, Gibson assembly was used to clone *3XFLAG-dCas9-tCYC1* in a *TRP1* integrative plasmid containing the promotor of the meiosis-specific gene *HOP1* (*pHOP1*; SGD coordinates 226,101-226,601; Chr. *IX*) to create *pHOP1-3XFLAG-dCas9-t**CYC1*. The plasmid containing *3XFLAG-dCas9/p**TEF1**p-t**CYC1* was a gift from Hodaka Fujii and obtained via Addgene.org (#62190; Addgene plasmid) (Fujita *et al.* 2018). Constructs that express different kinetochore subunits (*i.e.*, *CTF19*, *IML3*, *WIP1*, *CTF3*, and *NDC10*) were constructed by Gibson assembly. Yeast ORFs were PCR amplified from genomic (SK1) yeast DNA. All fusion constructs were cloned in the same order: *p**HOP1**-ORF-3xFLAG-dCAS9-tCYC1*. *DBF4* (PCR amplified from SK1 genomic DNA) was cloned COOH-terminally of *dCAS9*, and the two ORFs were separated by a 6×Glycine linker peptide. Constructs containing *ctf19*_*1–30*_, c*tf19*_*1–30(2x)*_, *ctf19**-9a*, and *ctf19*_*1–30 9A*_ were generated by Gibson assembly based on gene fragments synthesized by Genewiz. The two *ctf19*_*1–30*_ fragments in *ctf19*_*1–30(2x)*_ are separated by a 6xGlycine linker peptide. The *ctf19**-9A* allele is based on (Hinshaw *et al.* 2017), and carries the following mutations in *CTF19*: T4A, S5A, T7A, T8A, S10A, T13A, S14A, S16A, and S19A). SgRNA molecules were expressed from an URA3-integrated plasmid (pT040), which was a gift from John Wyrick and obtained via Addgene.org (#67640; Addgene plasmid) (Laughery *et al.* 2015). SgRNA expression was driven by the *pSNR52* For cloning of the three different sgRNA vectors used here, custom synthesized sgRNA cassettes for “mock,”, “*III*”, and “*VIII*” (Genewiz) were restriction cloned into pT040, to create the used *URA3* integrative plasmids. The used 20-mer target-specific complementary sequences (which are located directly upstream of a PAM sequence) were: “*III*”: 5′ TCT TAT ATA CAG GAG ATG GG 3′ (SGD coordinates: 209,871-209,890; Chr. *III*). “*VIII*”: 5′ AGA CCT TTA TAG TAC TGT TA 3′ (SGD coordinates: 146,203-146,222; Chr. *VIII*). All constructs were sequence verified.

For live cell reporter assays, we used two recombination reporter loci, as described in Vincenten *et al.* (2015). For the chromosome arm reporter, *p**YKL050c**-CFP* was integrated at the *THR1* locus; *pYKL050c**-RFP* was integrated at SGD coordinates 150,521-151,070; Chr. *VIII*; *p**YKL050c*-GFP* was introduced at the *ARG4* locus. For the centromeric reporter locus, *pYKL050c**-CFP* was integrated at the *THR1* locus; *p**YKL050c**-RFP* was integrated at *CEN8* (Chr. *VIII*); and *pYKL050c*-GFP* was introduced at SGD coordinates 115,024–115,582 (Chr. *VIII*). Plasmids containing *pYKL050c*-CFP/RFP/GFP* were described in Thacker *et al.* (2011).

For the recombination interval at chromosome *III*, a *HIS3* marker was integrated downstream of the *ARE1* gene (SGD coordinate: 214,010, Chr. *III*), using standard methodology. Diploids heterozygous for *ARE1*::*HIS3* allele were analyzed for genetic markers (*i.e.*, mating type and HIS+) by tetrad dissection followed by replica plating.

To generate SK1 strains carrying *ctf19**-9A* alleles, haploid strain yAM3563 (carrying *ctf19**Δ*::*KanMX6)* was transformed with PCR product amplified from plasmid AMp1619 and corresponding to full-length *ctf19**-9A* (carrying mutations: T4A, S5A, T7A, T8A, S10A, T13A, S14A, S16A, and S19A as previously described (Hinshaw *et al.* 2017) and a downstream marker (*LEU2*). G418-sensitive, leucine prototrophs carrying all mutations were confirmed by sequencing.

### Growth conditions

Solid and liquid yeast cultures were grown as described in Vincenten *et al.* (2015). With the exception of the *ndc10**-1* strains (Supplemental Material, Figure S2D), which were grown at 23°, all meiotic time courses were performed at 30°.

### SDS-PAGE and western blotting

Samples taken from synchronous meiotic cultures (5 ml; time points are indicted per experiments) were centrifuged at 2700 rpm for 3 min. Cell pellets were precipitated in 5 ml 5% TCA and washed with 800 µl acetone. Precipitates were dried overnight and resuspended in 200 µl protein breakage buffer (4 ml TE buffer, 20 µl 1 M DTT); 0.3 g glass beads were added and the cells in the samples were lysed using a FastPrep-24 (MP Biomedicals). Then, 100 µl of 3× SDS loading buffer was added, and processed using standard SDS-PAGE western blotting methodology. The following primary antibodies were used: α-Flag M2 (1:1000; Sigma-Aldrich), α-Flag (1:1000; Abcam,) α-HA (1:500, Biolegend, or 1:1000; Sigma-Aldrich), α-Pgk1 (1:1000; Thermo Fischer), α-GFP (1:1000; Roche).

### Co-immunoprecipitation

Samples taken from synchronous meiotic cultures (200 ml; samples were taken 5 hr post inoculation) were centrifuged at 2700 rpm for 3 min. Samples were resuspended in 500 µl M2 buffer [0.05 M Tris (pH 7.4), 0.15 M NaCl, 1% (v/v) Triton X-100, 1 mM EDTA] containing phenylmethylsulfonylfluoride, sodium orthovanadate, cOmplete Mini, EDTA free Protease Inhibitor Cocktail (Roche), and a protease inhibitor mix in DMSO (SERVA). Glass beads (0.6 g) were then added and the cells were lysed in a FastPrep-24 (MP Biomedicals). Lysates were sonicated using a BioruptorPlus (Diagenode) at 4° (set at 25 cycles of 25 sec). Lysates were centrifuged at 15,000 rpm (at 4° for 15 min); 450 µl of the cleared lysates were incubated with 1 µl of primary antibody [α-Flag M2 (1:400; Sigma-Aldrich)] at 4° for 3 hr; 25 µl of Protein G Dynabeads (Invitrogen-Thermo Fischer) was added and the samples incubated at 4° overnight. Resin was washed five times with 500 µl cold M2 buffer and once with 500 µl cold M2 buffer without detergent. Then, 50 µl of 2× SDS buffer was added and samples were heated at 65° for 30 min. For input, 50 µl of the clear supernatant was precipitated with 5 µl 100% TCA and washed with acetone. Precipitates were resuspended in 50 µl TCA resuspension buffer [7 M urea, 2% SDS, 50 mM Tris (pH 7.5)], and 25 µl of 3× SDS loading buffer were added. Samples were processed using standard SDS-PAGE western blotting methodology.

### Flow cytometry

Synchronous progression of meiotic cultures was assessed by flow cytometry as described in Vader *et al.* (2011), using an Accuri C6 Flow Cytometer (BD Biosciences).

### Fluorescent CO reporter assay

Diploid yeast strains carrying the fluorescent reporter construct were induced into synchronous meiotic liquid cultures. After 24 hr of incubation, 2 ml aliquots of those samples were lightly sonicated with a Sonifier 450 (Branson Ultrasonics Corporation) (tetrad integrity was not disrupted by sonication), spun down for 5 min at 4000 rpm, resuspended in 200 µl H_2_O, and mounted onto coverslides. Imaging was done using a Delta Vision Ultra High Resolution Microscope (GE Healthcare), whereby each chosen coordinates of the sample were imaged in the CFP, mCherry and Green channel. The pictures were processed with ImageJ. Only tetrads comprising four visible spores in the CFP channel were counted, in order to prevent confounding effects due to meiotic chromosome missegregation. Map distance (cM) and standard errors were calculated using online tools (http://elizabethhousworth.com/StahlLabOnlineTools/EquationsMapDistance.html). Statistical significance was calculated using Fisher’s exact test (https://www.socscistatistics.com/tests/fisher/default2.aspx).

### Chromatin immunoprecipitation

Cells of 100 ml sporulation culture (harvested 4.5 hr post inoculation) were crosslinked with 1% formaldehyde for 15 min at room temperature. Crosslinking was quenched for 5 min at room temperature by adding 2.5 M Glycine to a final concentration of 125 mM. Quenched cells were pelleted for 3 min at 4°, at 3000 rpm and washed once with 20 ml ice-cold 1× TBS buffer. Prechilled M2 lysis buffer and an equal volume of glass beads (Carl Roth) was added. Cells were lysed using a FastPrep-24 (MP Biomedicals). Cell lysates were mixed on a VXR basic Vibrax (IKA) for 2 min at 1500 rpm. Chromatin was fragmented by sonication using Branson Sonifier 450 at output control 2, constant cycle three times for 15 sec. In between runs, samples were kept on ice for 2 min. Cellular debris was pelleted for 10 min at 4°, 15,000 rpm and crude lysate was collected. As input sample, 50 μl of the crude lysate was added to 200 μl of 1× TE/1% SDS buffer and stored at 4° until reversal of crosslinking. For α-Flag ChIPs, 500 μl of the crude lysate was incubated with 40 μl of 50% slurry of α-Flag M2 beads (Sigma-Aldrich) for 2 hr, after which resin was washed four times with 500 μl of ice-cold M2 buffer and once with 500 μl of M2 buffer without detergent. Protein–DNA complexes were eluted from the beads by adding 200 μl of ice-cold M2 buffer without detergent containing 3×FLAG peptides (Sigma-Aldrich) (final concentration of 150 ng/μl) and rotated at 4° for 30 min. Resin was pelleted in a refrigerated centrifuge for 30 sec at 9000 rpm and the supernatant containing the protein-DNA complexes was transferred to a new tube. This step was repeated and 800 μl of 1× TE/1% SDS buffer was added to the total eluate. For α-HA ChIPs, 500 μl of the crude lysate was incubated with 1 μl of α-HA antibody (BioLegend) for 3 hr at 4°; 35 μl of a 50% slurry of protein G Dynabeads (Invitrogen) was added, and lysate was incubated overnight at 4°. Resin was washed four times with ice-cold M2 buffer without inhibitor, and once with ice-cold M2 buffer without detergent. Supernatant was removed, and resin was resuspended in 200 μl of 1× TE/1% SDS buffer and incubated at 65° for 18 hr to reverse crosslinking. Glycogen (5 μl; 20 mg/ml) and proteinase K (5 μl; 20 mg/ml; Roche) were added to the samples and incubated at 37° for 2 hr. ChIP samples were split, and 68.7 μl of 3 M LiCl and 1 ml of 100% ethanol was added to the input and ChIP samples and precipitated at –20° overnight. DNA was pelleted at 15,000 rpm for 10 min and washed once with 75% ethanol. DNA pellets were resuspended in 50 μl of TE containing RNAse A (1/100 μl) and incubated at 37° for 30 min. Real time quantitative PCR (qPCR) was performed using a 7500 Fast Real-Time PCR System (Applied Biosystems). PerfeCTa SYBR Green FastMix was used. The threshold cycle number (C_t_ value) of a fast two-step cycling program for product detection was used to normalize the ChIP-qPCR data according to the Percent Input method.

### Primers used

GV2678: 5′ GCT AGG CGC GGG TTC TGT GG 3′
GV2680: 5′ CAT CAC TAC GGG AAA CCA AA 3′; primer pair amplifies SGD coordinates 209,913-209,772; Chr. *III. (YCRO47C)*
GV2472: 5′ TAA ATG TAC CTT ACC ATG TTG 3′
GV2473: 5′ TCC GGA CTC GTC CAA TCT TT 3′; primer pair amplifies SGD coordinates 146,165-146,236; Chr. *VIII*.
GV2569: 5′ GAT CAG CGC CAA ACA ATA TGG AAA ATC C 3′
GV2570: 5′ AAC TTC CAC CAG TAA ACG TTT CAT ATA TCC 3′; primer pair amplifies SGD coordinates 114,321-114,535; Chr. *III (**CEN3**)*.

Primer efficiencies (calculated using standard procedures) were as follows: (GV2678/GV2680: 2,04; GV2472/GV2473: 2,28; GV2569/GV2570: 2,16)

### Southern blot analysis of DSB formation

Southern blotting was performed as previously described (Vader *et al.* 2011), using the following probe (SGD coordinates): *YCR047C*; 209,361-201,030; Chr. *III*. DSB intensities were calculated from three independent experiments using ImageJ. Error bars indicate SEM.

### Spo11-oligo mapping

Spo11 oligo mapping data from wild-type strains mapped to the S288c genome assembly R64 (sacCer3) and normalized to the total number of uniquely mapped reads (reads per million) was retrieved from the Gene Expression Omnibus (GEO), access number: GSE67910 (GSM1657849 and GSM1657850) (Zhu and Keeney 2015). Peaks were visualized on Integrative Genome Browser.

### Data availability

Strains and plasmids are available upon request. The authors affirm that all data necessary for confirming the conclusions of the article are present within the article, figures, and tables. Supplemental material available at figshare: https://doi.org/10.25386/genetics.12855311.

## Results

**Figure 1:**
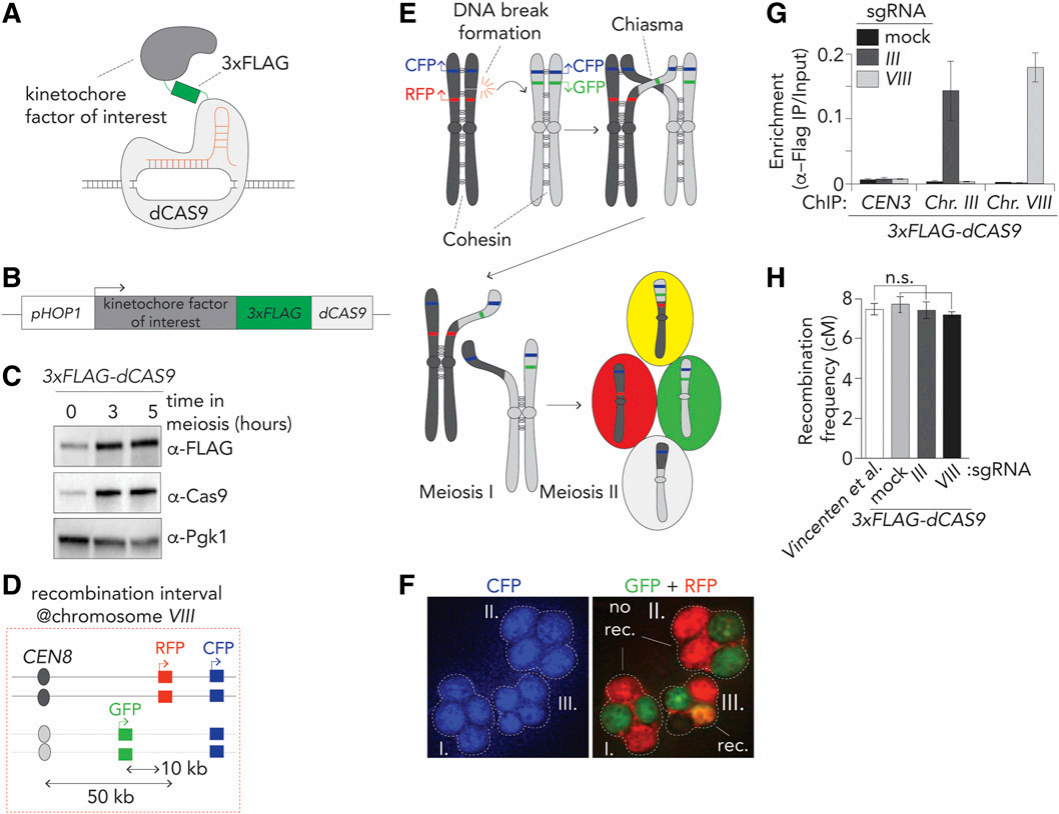
a dCas9/CRISPR-based targeting system. (A) Schematic of dCas9-based fusion protein used in this study. Note that the 3×Flag moiety also functions as a peptide linker in between the kinetochore factor of interest and dCas9. (B) Schematic of fusion construct design. (C) Western blot analysis of expression of 3×Flag-dCas9 during meiotic G2/prophase at defined hours after induction into the meiotic program. Pgk1 was used as a loading control. (D) Schematic of live cell reporter assay on the right arm of Chromosome *VIII*. See *Materials and Methods* for more information. (E) Schematic of meiotic recombination, chromosome segregation, and assortment of chromosomes in haploid gametes, yielding differentially fluorescent behaviors that report on recombination frequencies. (F) Example of three tetrads from a meiotic culture with the described live cell reporter. Cells I. and II. are parental ditype, III. is tetratype. No rec, no recombination; rec, recombination. (G) ChIP-qPCR (α-Flag ChIP) analysis of CEN3/Chr. *III*/Chr.*VIII* regions in yeast strains expressing 3×Flag-dCas9 in combination with sgRNA “*mock*,” “*III*” and “*VIII*” during meiotic G2/prophase (5 hr). Primers pairs used for *CEN3*: GV2569/G2570, *III*: GV2464/GV2465, *VIII*: GV2472/GV2473. Average of three experiments. (H) Map distances in centiMorgans (cM) and standard error determined for chromosomal arm interval as described in *Materials and Methods* and depicted in (D). Data are from (Vincenten *et al.* 2015) and for 3×Flag-dCas9 in combination with sgRNAs “*mock*,” “*III*” and “*VIII*”, as indicated. *P*-values were obtained using Fisher’s exact test (n.s., nonsignificant ≥0.05, *P < 0.05; **P < 0.0001). See Table S1 for raw data and number of cells counted.

To dissect contributions of kinetochore factors to regulation of meiotic recombination, we developed a system to query the roles of kinetochore (and specifically, Ctf19c) subunits in directing meiotic chromosome fragmentation and repair. We were inspired by earlier approaches that relied on integration of ectopic DNA arrays coupled to the expression of cognate targeting units fused to genes of interest, to isolate aspects of kinetochore function (Kiermaier *et al.* 2009; Lacefield *et al.* 2009; Gascoigne *et al.* 2011; Ho *et al.* 2014). However, since DNA integration can cause unwanted effects on meiotic DSB/recombination patterns, we opted for an approach not requiring integration of foreign DNA at a locus of interest. The CRISPR-dCas9 system (Wang *et al.* 2016) employs a mutated, catalytically dead, version of Cas9 nuclease (Gilbert *et al.* 2013) (dCas9) that can be recruited to genomic loci when paired with specific single guide RNAs (sgRNAs) (Figure 1A). sgRNA-driven dCas9 recruitment occurs without cleavage of the targeted DNA sequence, and can direct fused proteins of interest to defined loci. This approach has been used for myriad applications (*e.g.*, Xu *et al.* 2016; Liu *et al.* 2017). We used a dCas9 that was tagged at its NH_2_-terminus with a 3×Flag tag (3×Flag-dCas9) and placed under the control of the promoter of the meiosis-specific *HOP1* gene (*pHOP1*, creating *pHOP1-3XFLAG-dCas9*), ensuring meiosis-specific expression to avoid potential interference with chromosome segregation during vegetative growth (Vershon *et al.* 1992) (Figure 1, A and B). Western blot analysis using α-Cas9 or α-Flag confirmed the meiosis-specific induction of 3×Flag-dCas9 (Figure 1C). We combined this system with a fluorescence-based assay to measure local CO recombination frequencies within a region on the (nonpericentromeric) arm of chromosome *VIII* (Thacker *et al.* 2011; Vincenten *et al.* 2015) (Figure 1, D–F). Throughout this study, we used three sgRNA cassettes (Laughery *et al.* 2015) in combination with dCas9-fusion constructs: one sgRNA targets an intergenic chromosomal position between the genes *YHR020W* and *YHR021C* within the 10 kb interval flanked by the *GFP* and *RFP* markers of the recombination reporter on chromosome *VIII* (Thacker *et al.* 2011) (Figure 1D and Figure S1A). We previously observed a ∼6 kb sized DSB effect surrounding centromeres (Vincenten *et al.* 2015), and chose a sequence positioned ∼2.5 kb from the major DNA break hotspot in the divergent promoters of the genes *YHR019C* and *YHR020W* (Pan *et al.* 2011). This sgRNA molecule is referred to as “*VIII*”. We also used a sgRNA (“*III*”), which directs the dCas9 to the intergenic region in between *YCR045C* and *YCR046C* on chromosome *III*, in the vicinity (∼1.8 kb away) of a strong natural DSB hotspot (“*YCR047C*”; see Figure 6A and see below). A sgRNA molecule that lacks the 20-nt target sequence, referred to as “mock,” was used as control. sgRNA *VIII* and *III* are located in intergenic regions to minimize interference with gene expression in order to prevent potential indirect effects on DSB activity (Figure 6 and Figure S1). α-Flag ChIP-qPCR confirmed specific enrichment of 3×Flag-dCas9 when combined with the corresponding sgRNAs (Figure 1G). We ascertained that targeting of 3×Flag-dCas9 within the interval on chromosome *VIII* or when combined with *III* or mock sgRNAs did not interfere with recombination frequencies (Figure 1H and Table S1). Indeed, upon 3×Flag-dCas9 targeting, observed crossover frequencies were indistinguishable from reported frequencies within this interval (Vincenten *et al.* 2015). These results verify the development of our ectopic targeting system to investigate meiotic recombination, and show that dCas9 can be targeted to defined regions within the genome without causing unwanted effects on meiotic recombination frequencies.

**Figure 2:**
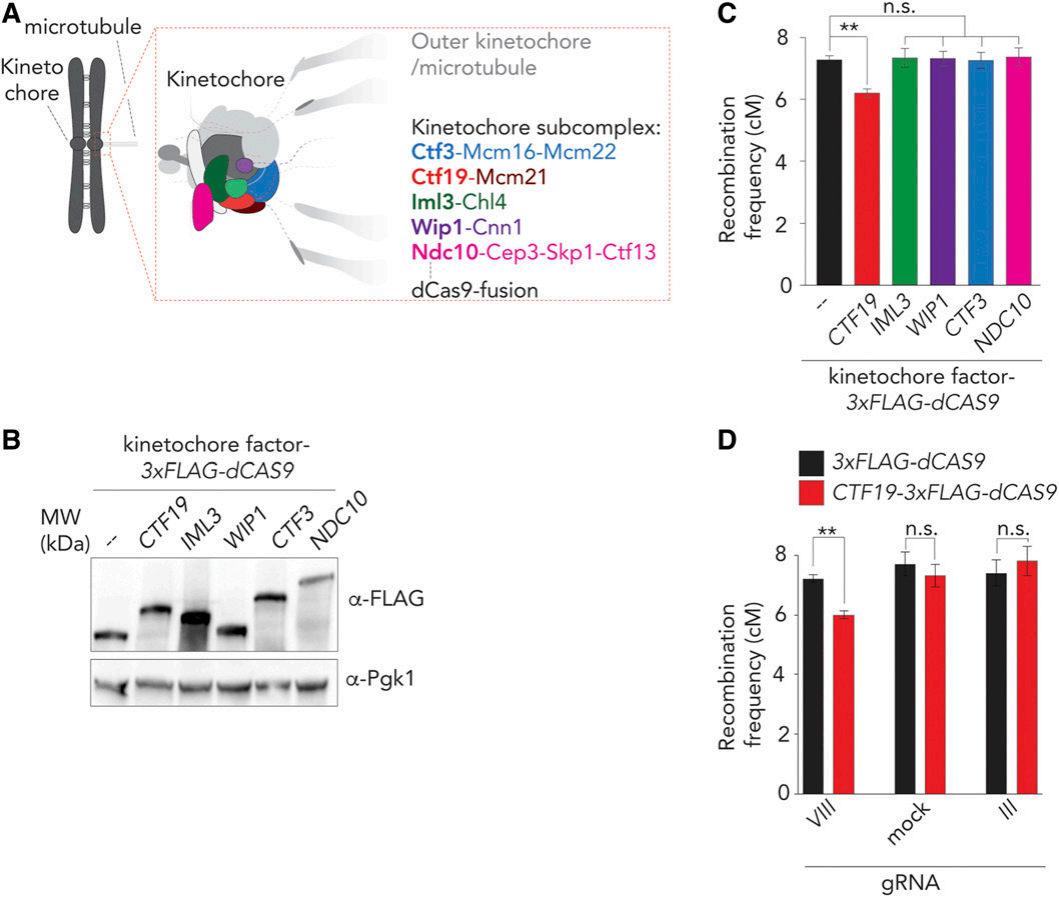
dCas9/CRISPR-based targeting reveals a role for Ctf19 in crossover (CO) control. (A) Schematic of the budding yeast kinetochore, adapted from Hinshaw and Harrison (2019). The investigated kinetochore subcomplexes are highlighted. Individual factors that were used as dCas9-fusions are indicated in bold. (B) Western blot analysis of expression of indicated 3×FLAG-dCas9 fusion constructs during meiotic G2/prophase (5 hr). Pgk1 was used as a loading control. Representative of three experiments. (C) Map distances in centiMorgans (cM) and standard error determined for chromosomal arm interval in cells expressing indicated 3×FLAG-dCas9 fusion constructs and “*VIII*” sgRNA. *P*-values were obtained using Fisher’s exact test (n.s., nonsignificant ≥0.05, *P < 0.05; **P < 0.0001). See Table S1 for raw data and number of cells counted. D. Map distances in cM and standard error determined for chromosomal arm interval in cells expressing indicated 3×FLAG-dCas9 fusion constructs and “*mock*,” “*III*,” or “*VIII*” sgRNAs. *P*-values were obtained using Fisher’s exact test (n.s. ≥0.05, *P < 0.05; **P < 0.0001). See Table S1 for raw data and number of cells counted.

Fusions of dCas9 were generated in order to interrogate contributions of selected kinetochore factors (and directly associated and cotargeted factors) to suppression of meiotic recombination. We fused factors of the budding yeast kinetochore at their COOH-termini with the 3×Flag-dCas9 moiety (*i.e.*, the organization of these polypeptides is: protein of interest-3×Flag-dCas9, where the 3×Flag moiety also functions as an unstructured linker peptide). Functional COOH-terminal GFP fusions of the factors we used here have been described (*e.g.*, Ho *et al.* 2014; Schmitzberger *et al.* 2017; Schleiffer *et al.* 2012), which we reasoned increased the chances that our dCas9 fusions would be functional. Five factors that represent kinetochore/Ctf19C subcomplexes within the Ctf19C were investigated: Ctf19, Iml3, Wip1, Ctf3, and Ndc10 (Figure 2A). All factors were efficiently expressed during meiosis when fused to 3×Flag-dCas9 (Figure 2B). Expression via *p**HOP1* ensures induction of these fusion genes in early meiosis.

Importantly, Ctf19-, Iml3-, Wip1-, Ndc10-, and Ctf3-3xFlag-dCas9 fusions rescued spore viability defects observed in their respective gene deletions/temperature-sensitive mutant (Figure 2, A–D and Table S2), confirming functionality. In addition, ectopic expression in a wild-type background did not interfere with meiotic chromosome segregation (Figure S2, A–D).

We investigated whether ectopic recruitment of these factors resulted in effects on recombination frequencies on chromosome *VIII*. Interestingly, we observed a moderate, but significant, reduction in recombination frequency (within the interval on chromosome *VIII*) in cells expressing Ctf19-3×Flag-dCas9 in combination with sgRNA *VIII* (Figure 2C). This effect was specific for Ctf19: targeting Iml3, Wip1, Ctf3 or Ndc10 did not significantly change frequencies. The Ctf19-driven effect depended on its local recruitment: when *p**HOP1**-**CTF19**-3XFLAG-dCAS9* was combined with mock or *III* sgRNAs, no changes on recombination frequencies were observed (Figure 2D). These data demonstrate the feasibility of our dCas9-targeting system and isolate the Ctf19 subunit of the kinetochore as a factor whose local targeting at a noncentromeric locus can influence meiotic recombination.

**Figure 3:**
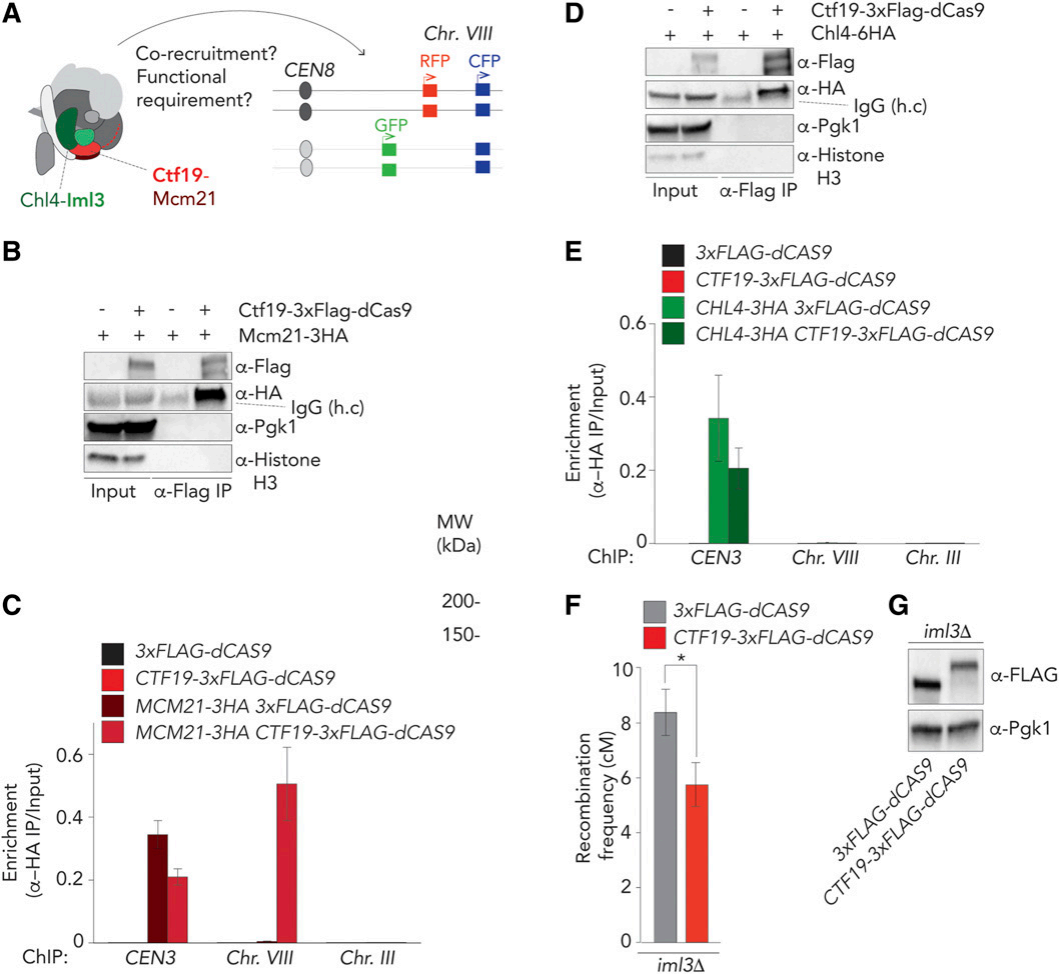
Dissection of Ctf19-dependent crossover (CO) control. (A) Schematic of the budding yeast kinetochore, adapted from Hinshaw and Harrison (2019), indicating effects of Ctf19-targeting on chromosome arm interval (*i.e.*, cotargeting of binding partners and functional requirement). (B) Co-immunoprecipitation of Ctf19-3×Flag-dCas9 and Mcm21-3HA (via α-Flag IP) during meiotic prophase (5 hr into meiotic program). Pgk1 and Histone H3 are used as loading controls. Representative of three experiments. (C) ChIP-qPCR (α-HA ChIP) analysis of *CEN3*/Chr. *III/*Chr. *VIII* regions in yeast strains expressing indicated factors (5 hr). Primers pairs used for *CEN3*: GV2569/G2570, *III*: GV2464/GV2465, *VIII*: GV2472/GV2473. Average of three experiments. (D) Co-immunoprecipitation of Ctf19-3×Flag-dCas9 and Chl4-6HA (via α-Flag IP) during meiotic prophase (5 hr into meiotic program). Pgk1 and Histone H3 are used as loading control. Representative of three experiments. (E) ChIP-qPCR (α-HA ChIP) analysis of *CEN3*/Chr. *III/*Chr. *VIII* regions in yeast strains expressing indicated factors (5 hr). Primers pairs used for *CEN3**:* GV2569/G2570, *III*: GV2464/GV2465, *VIII:* GV2472/GV2473. Average of three experiments. (F) Map distances in centiMorgans (cM) and standard error determined for chromosomal arm interval in *iml3**Δ* cells expressing indicated 3×FLAG-dCas9 fusion constructs and “*VIII*” sgRNA. *P*-values were obtained using Fisher’s exact test (n.s., nonsignificant ≥0.05, *P < 0.05; **P < 0.0001). See Table S1 for raw data and number of cells counted. (G) Western blot analysis of expression of indicated 3×FLAG-dCas9 fusion constructs in *iml3**Δ* cells during meiotic G2/prophase (5 hr), as used in (F) Representative of three experiments.

Ctf19 is an RWD domain-containing protein that forms a stable heterodimer with Mcm21, also an RWD domain protein (Schmitzberger and Harrison 2012). Together with Ame1 and Okp1, the Ctf19-Mcm21 dimer forms the COMA Ctf19c-subcomplex (De Wulf *et al.* 2003) (Figure 3A). We found that the fusion protein Ctf19-3×Flag-dCas9 co-immunoprecipitates with Mcm21-3HA (Figure 3B), and was able to corecruit Mcm21-3HA to the target locus on chromosome *VIII* (Figure 3C). Thus, Ctf19-Mcm21 (and possibly the entire COMA complex) is corecruited upon targeting of Ctf19 to an ectopic location. The assembly of additional Ctf19-C proteins, such as the Chl4-Iml3 subcomplex, at kinetochores depends on COMA (Schmitzberger *et al.* 2017) (Pot *et al.* 2003). Despite an efficient interaction between Ctf19-3×Flag-dCas9 and Chl4-3HA [as judged by co-immunoprecipitation (Co-IP); Figure 3D], we did not observe Chl4-3HA accumulation at the target locus on arm *VIII* in *p**HOP1**-**CTF19**-3XFLAG-DCAS9*, *sgRNA-VIII* expressing cells. Thus, ectopic targeting of Ctf19 is not sufficient to corecruit the Chl4-Iml3 complex (Figure 3E and Figure 3SA). The discrepancy between the interaction and recruitment could be explained by the observed interaction taking place at native kinetochores, where Ctf19-3×Flag-dCas9 is present, in addition to the ectopic targeting site (Ctf19-dCas9 rescued *ctf19**Δ*, indicating incorporation into kinetochores, Figure S2A). No interaction between Mtw1-GFP (a non-Ctf19C kinetochore factor) and Ctf19-3×Flag-dCas9 (Figure S3B) was detected. These data demonstrate that ectopic targeting of Ctf19 leads to corecruitment of its direct binding partner Mcm21, but is insufficient to lead to corecruitment of other Ctf19C/kinetochore factors, such as Iml3-Chl4 and Mtw1.

Our results suggest that the effect of Ctf19-3×Flag-dCas9 on crossover suppression is encoded within the factors that are recruited to the ectopic site. Thus, the Ctf19-driven effect should occur independently of nonrecruited factors, such as the Chl4-Iml3 complex. Indeed, targeting of Ctf19-3×Flag-dCas9 in *iml3**Δ* cells led to an equal reduction in recombination rates as in a wild-type background (Figure 3, F and G). This points to a central role for Ctf19 (and potentially its associated COMA complex binding partners, such as Mcm21) in regulating CO suppression.

**Figure 4:**
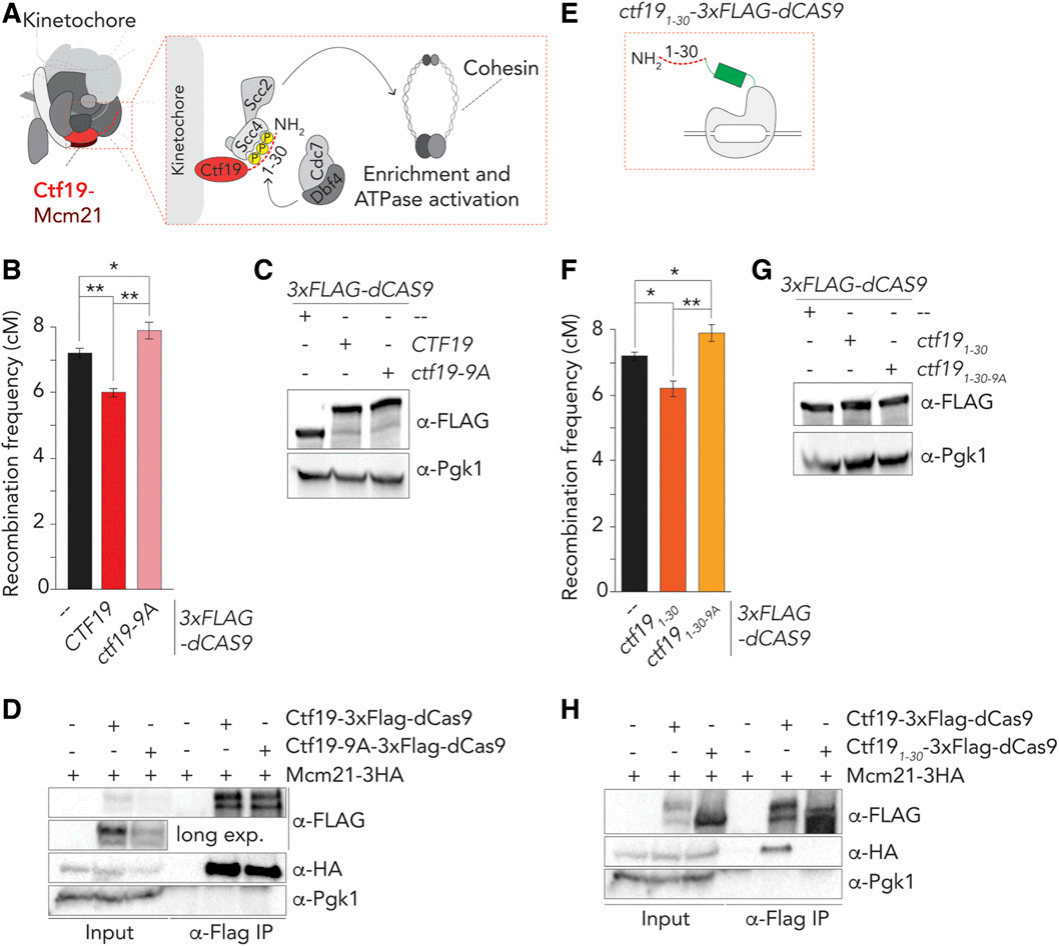
Phosphorylation of the NH_2_-terminus of Ctf19-dependent drives crossover (CO) control. (A) Schematic of the budding yeast kinetochore, adapted from Hinshaw and Harrison (2019), indicating the molecular connection between DDK, the NH_2_-terminus of Ctf19, Scc2/Scc4, and cohesin function. (B) Map distances in centiMorgans (cM) and standard error determined for chromosomal arm interval in cells expressing indicated 3×FLAG-dCas9 fusion constructs and “*VIII*” sgRNA. *P*-values were obtained using Fisher’s exact test (n.s., nonsignificant ≥0.05, *P < 0.05; **P < 0.0001). See Table S1 for raw data and number of cells counted. (C) Western blot analysis of expression of indicated 3×FLAG-dCas9 fusion constructs cells during meiotic G2/prophase (5 hr), as used in (B) Representative of three experiments. (D) Co-immunoprecipitation of Ctf19-3×Flag-dCas9, Ctf19-9A-3×Flag-dCas9 and Mcm21-3HA (via α-Flag IP) during meiotic prophase (5 hr into meiotic program). Pgk1 and Histone H3 are used as loading control. Representative of three experiments. (E) Schematic of Ctf19_1-30_-3×Flag-dCas9. (F) Map distances in centiMorgans (cM) and standard error determined for chromosomal arm interval in cells expressing indicated 3×FLAG-dCas9 fusion constructs and “*VIII*” sgRNA. *P*-values were obtained using Fisher’s exact test (n.s. ≥0.05, *P < 0.05; **P < 0.0001). See Table S1 for raw data and number of cells counted.(G). Western blot analysis of expression of indicated 3×FLAG-dCas9 fusion constructs cells during meiotic G2/prophase (5 hr), as used in (F). Representative of three experiments. H. Co-immunoprecipitation of Ctf19-3XFlag-dCas9, Ctf19_1–30_-3XFlag-dCas9 and Mcm21-3HA during meiotic prophase (5 hr into meiotic program). Pgk1 is used as loading control. Representative of three experiments.

To dissect how Ctf19 influences meiotic recombination, we focused on the role of Ctf19 in regulating cohesin (Fernius and Marston 2009; Hinshaw *et al.* 2015, 2017) (Figure 4A). Ctf19 recruits Scc2-Scc4, a regulator of chromosomal loading and stimulator of cohesin ATPase activity, to kinetochores, and influences cohesin throughout pericentromeres (Fernius and Marston 2009; Hinshaw *et al.* 2015, 2017; Petela *et al.* 2018; Davidson *et al.* 2019; Gutierrez-Escribano *et al.* 2019). Scc2-Scc4 associates with the 30 NH_2_-terminal amino acids of Ctf19, in a manner that is dependent on phosphorylation of nine serine/threonine residues by the Cdc7/Dbf4 kinase (also known as DDK) (Hinshaw *et al.* 2017). Mutating these residues to nonphosphorylatable residues (in the *ctf19**-9A* allele) impairs efficient recruitment of Scc2-Scc4 and affects cohesin function (Hinshaw *et al.* 2017). When targeted to the target locus on arm *VIII*, Ctf19-9A was unable to suppress recombination frequencies (in fact, CO frequency was slightly increased under this condition) (Figure 4, B and C). Ctf19-9A was still able to associate with Mcm21 and Chl4 (Figure 4D and Figure S4A). These results suggest that the effect of Ctf19 on CO suppression was likely connected to its role in kinetochore-recruitment of Scc2-Scc4, and effects on cohesin function.

We aimed to explore this idea further. First, we tested the ability of a construct containing the first 30 NH_2_-terminal amino acids of Ctf19 (which fall outside of the structured RWD) in mediating CO reduction. Strikingly, we found that the first 30 NH_2_-terminal amino acids of Ctf19 (when fused to dCas9) were sufficient to instigate CO suppression to the same level as full length Ctf19 (Figure 4, E–G). Importantly, as in the full-length case, this suppression was abolished upon mutation of the 9 DDK-targeted residues in this NH_2_-terminal fragment. Ctf19_1–30_ was unable to associate with Mcm21 or Chl4, as expected from the described requirement for the RWD domain of Ctf19 in mediating interactions with the COMA and Ctf19c components (Figure 4H and Figure S4B) (Schmitzberger and Harrison 2012; Schmitzberger *et al.* 2017). Thus, suppression of meiotic recombination instated by Ctf19 can be provided by its NH_2_-terminal tail, and depends on residues important for the recruitment of the Scc2-Scc4 cohesin regulator.

**Figure 5:**
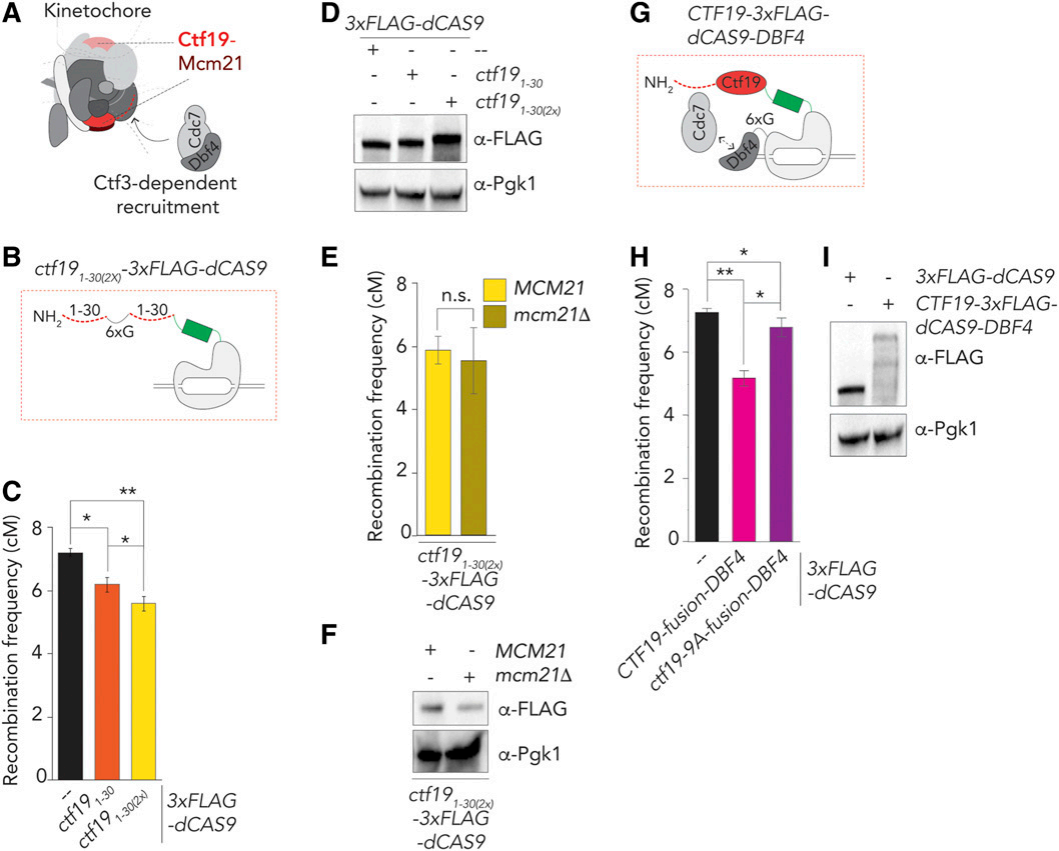
Manipulating Ctf19-dependent crossover (CO) strength. (A) Schematic of the budding yeast kinetochore, adapted from Hinshaw and Harrison (2019), indicating the “dimeric” nature of Ctf19c within the kinetochore, and the role of Ctf3 in DDK recruitment. (B) Schematic of Ctf19_1-30(2×)_-3×Flag-dCas9. 6×G indicates 6×Glycine present between the two Ctf19 moieties. (C) Map distances in centiMorgans (cM) and standard error determined for chromosomal arm interval in cells expressing indicated 3×FLAG-dCas9 fusion constructs and “*VIII*” sgRNA. *P*-values were obtained using Fisher’s exact test (n.s., nonsignificant ≥0.05, *P < 0.05 **P < 0.0001). See Table S1 for raw data and number of cells counted. (D) Western blot analysis of expression of indicated 3×FLAG-dCas9 fusion constructs cells during meiotic G2/prophase (5 hr), as used in (C). Representative of three experiments. (E) Map distances in cM and standard error determined for chromosomal arm interval in cells expressing Ctf19_1-30(2×)_-3×Flag-dCas9 and “*VIII*” sgRNA in *MCM21* or *mcm21**Δ* cells. *P*-values were obtained using Fisher’s exact test (n.s. ≥0.05, *P < 0.05; **P < 0.0001). See Table S1 for raw data and number of cells counted. (F) Western blot analysis of expression of ctf19_1-30)(2×)_-3×Flag-dCas9 in *MCM21* or *mcm21**Δ* cells during meiotic G2/prophase (5 hr), as used in (E). Representative of three experiments. (G) Schematic of Ctf19-3×Flag-dCas9-Dbf4. 6×G indicates 6×Glycine present between the dCas9 and Dbf4. (H) Map distances in cM and standard error determined for chromosomal arm interval in cells expressing indicated 3×FLAG-dCas9 fusion constructs and “*VIII*” sgRNA. *P*-values were obtained using Fisher’s exact test (n.s. ≥0.05, *P < 0.05; **P < 0.0001). See Table S1 for raw data and number of cells counted. (I) Western blot analysis of expression of indicated 3×FLAG-dCas9 fusion constructs cells during meiotic G2/prophase (5 hr), as used in (H). Representative of three experiments.

Although our recombination analysis established that ectopic targeting of Ctf19 causes CO suppression, the observed effect was not as strong as (Ctf19-dependent) suppression of recombination at native pericentromeres (Vincenten *et al.* 2015). This can ostensibly be because certain aspects/factors of kinetochores that contribute to recombinational suppression might not be (efficiently) recapitulated in our targeting system. We aimed to address this possibility. First, we considered the stoichiometry of the kinetochore. It is assumed that the kinetochore contains two Ctf19c assemblies (Hinshaw and Harrison 2019; Yan *et al.* 2019) (Figure 5A). In our dCas9-targeting system, we target a single Ctf19-molecule; we thus engineered a fusion that allowed “dimeric” targeting of Ctf19. We made use of the fact that Ctf19_1-30_ was sufficient to trigger CO suppression. We constructed a dimeric Ctf19_1–30_ (Ctf19_1–30(2X)_)-dCas9 fusion (Figure 5B), and expression of this construct led to a stronger reduction on recombination frequency as compared to the “monomeric” Ctf19_1–30_ (Figure 5, C and D). Suppression of crossover activity in this “dimeric” construct was present even in *mcm21**Δ* cells (Figure 5, E and F), strengthening the conclusion that CO suppression is driven by the NH_2_ terminus of Ctf19, and occurs independently of the binding partner of Ctf19, Mcm21.

Next, we focused on Cdc7/DDK, which is recruited to kinetochores in a Ctf3-dependent manner (Hinshaw *et al.* 2017). DDK is responsible for the phosphorylation-dependent binding of Scc2-Scc4 to the NH_2_-terminus of Ctf19 (Hinshaw *et al.* 2017). We surmised that Ctf3 (and thus DDK) would not be corecruited by Ctf19-dependent targeting. Under such an assumption, nonkinetochore, chromatin-associated DDK would be responsible for (potentially inefficient) phosphorylation of Ctf19. Cdc7/DDK is associated with traveling replisomes (Takahashi *et al.* 2008; Murakami and Keeney 2014), and this pool of DDK could be responsible for phosphorylation of targeted Ctf19. We aimed to corecruit Dbf4 (and with it Cdc7) to Ctf19. We generated a *CTF19**-dCAS9-**DBF4* construct, wherein Dbf4 is fused to the COOH-terminus of dCas9 (note that, in this construct, dCas9 and Dbf4 are separated by an linker peptide) (Figure 5G). Interestingly, expressing this chimeric fusion construct led to stronger suppression of crossover frequency as compared to Ctf19-dCas9 (Figure 5, H and I). Importantly, mutation of the nine NH_2_ phosphoacceptor sites of Ctf19 in a fusion between Ctf19, dCas9 and Dbf4 (*i.e.*, *ctf19**-9A-dCAS9-**DBF4*) largely eliminated CO suppression (Figure 5H). These data suggest that efficient phosphorylation of the NH_2_ terminus of Ctf19, driven by DDK, is crucial for CO suppression.

**Figure 6:**
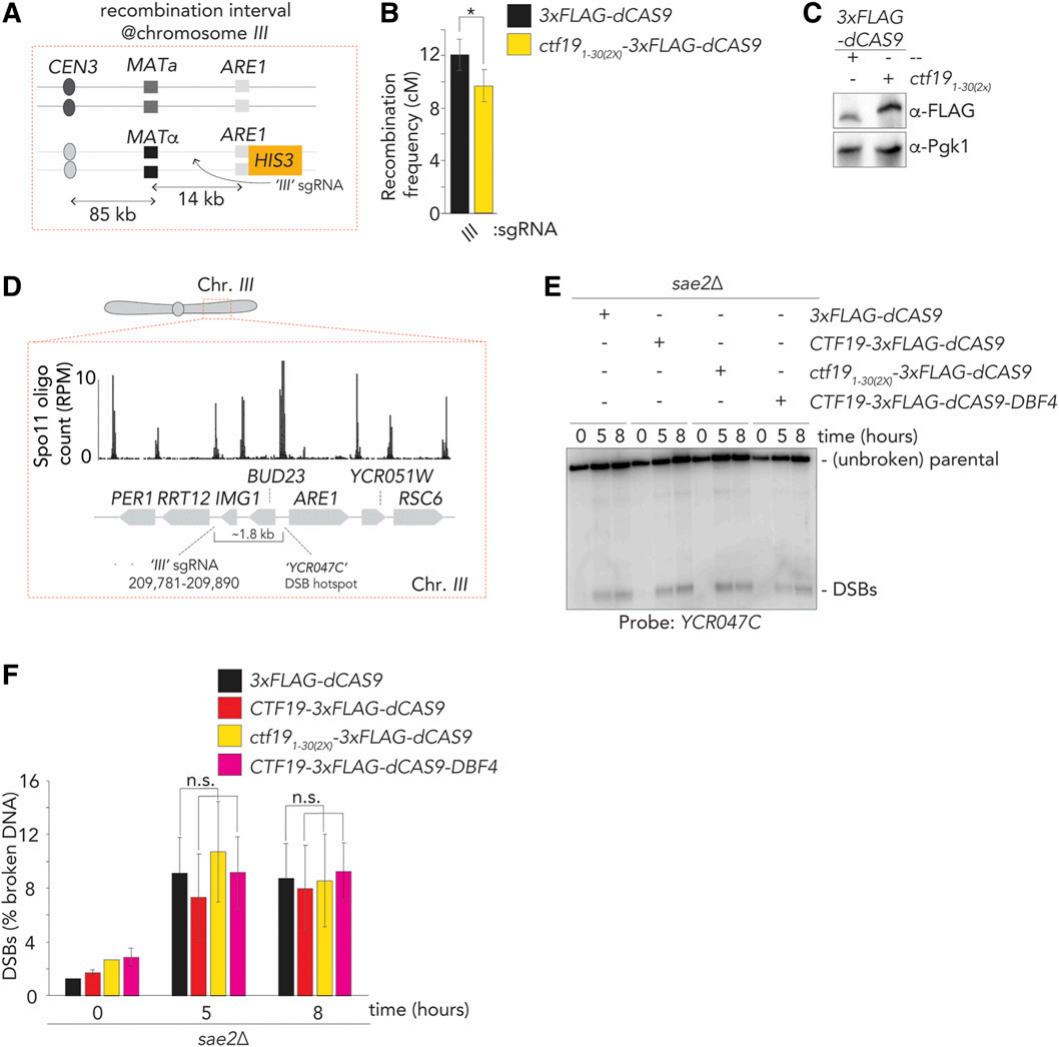
DSBs are not affected by dCas9-dependent targeting of Ctf19 fusions. (A) Schematic of Chromosome III recombination interval. (B) Map distances in centiMorgans (cM) and standard error determined for Chromosome III interval as in (A), in 3×Flag-dCas9 and Ctf19_1-30(2×)_-3×Flag-dCas9 and “*III*” sgRNA as determined by tetrad analysis . *P*-value was obtained using Fisher’s exact test (n.s., nonsignificant ≥0.05, *P < 0.05; **P < 0.0001). (C) Western blot analysis of expression of 3×Flag-dCas9 and ctf19_1-30)(2×)_-3×Flag-dCas9 during meiotic G2/prophase (5 hr), as used in (B) Representative of three experiments. (D) Schematic of the genomic region around the “*YCR047C*” DSB hotspot on Chromosome *III*. SGD coordinates for binding of sgRNA “*III*” are indicated. Representative genome browser profile of meiotic hotspots for Spo11-oligo mapping (Zhu and Keeney 2015). Normalized Spo11 oligo counts (RPM) is shown. (E) Southern blot of YCR047C DSB hotspot, in yeast expressing the indicated dCas9 constructs and the sgRNA “*III*”. Time into the meiotic time course is indicated. Note that the *sae2**Δ* background was used to prevent DSB resection and repair. Representative of three experiments. (F) Quantification of (E); error bars indicate SEM from three experiments.

We aimed to investigate (i) whether CO suppression driven by Ctf19 could also be transplanted to another genomic locus, and (ii) how ectopic targeting led to local CO suppression. To test these two questions, we used our sgRNA *III* to direct dCas9 fusions to a site in between the *MATa/α* locus and *ARE1*, on the right arm of chromosome *III* (Figure 6, A and D, see also Figure 1G). We used tetrad dissection to query CO frequencies within this interval, in cells expressing dCas9 or Ctf19_1–30(2X)_-dCas9. Expressing the Ctf19 dimeric construct triggered a significant reduction in CO frequency within this genomic region (Figure 6, B and C). We earlier proposed that CO suppression at pericentromeres is achieved by (i) DSB suppression and (ii) a channelling of remaining DSBs into a repair pathway that yields intersister CO repair over interhomolog CO repair (Vincenten *et al.* 2015). Ctf19 likely affects both pathways (Vincenten *et al.* 2015), and we investigated whether targeting of Ctf19 led to decreases local DSB activity. We recruited several Ctf19-fusion constructs to the vicinity of the *YCR047C* DSB hotspot (which lies within the *MATa**/α-**ARE1* interval on chromosome *III*), using sgRNA *III*. As shown in Figure 6, D–E, the targeting of either Ctf19, Ctf19_1-30(2X)_, or Ctf19 together with Dbf4, did not significantly alter DSB levels, as judged by Southern blot analysis. Thus, the CO-suppressive functionality seen in the Ctf19-based targeting modules likely occurs independently of a DSB-reducing effect. We suggest that the DSB-protective role of Ctf19/Ctf19c is related to its structural role in establishing kinetochore integrity (Pot *et al.* 2003) (Pekgoz Altunkaya *et al.* 2016; Lang *et al.* 2018).

**Figure 7:**
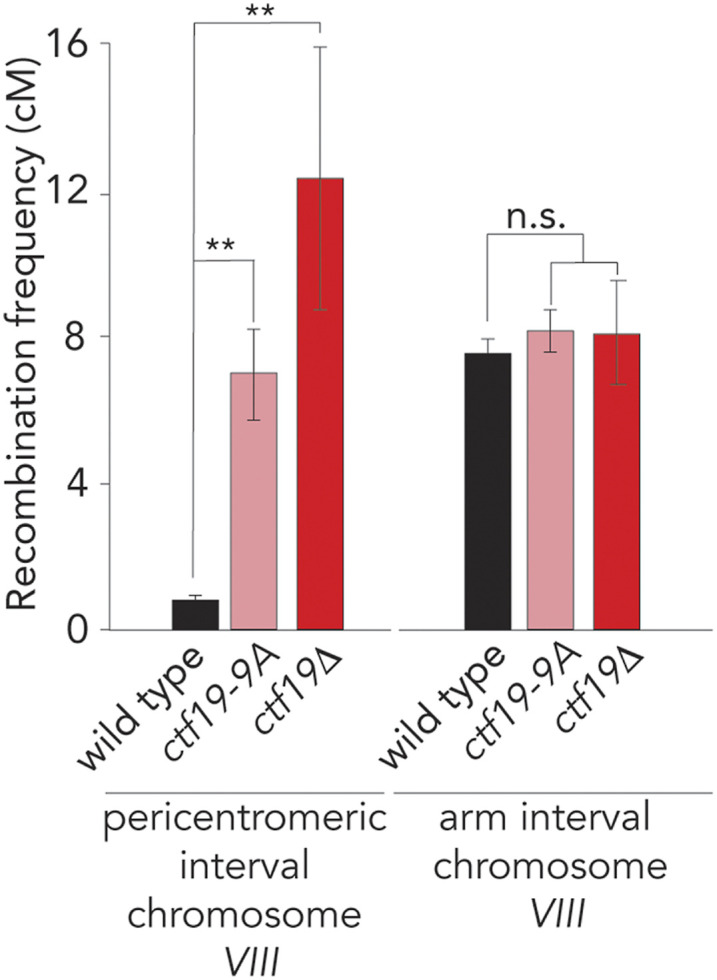
The DDK-Ctf19-Scc2/Scc4-cohesin pathway affects pericentromeric crossover (CO) suppression. (A) Map distances in centiMorgans (cM) and standard error determined for a pericentromeric (left panel) and chromosomal arm (right panel) intervals in wild type, *ctf19**-9A*, and *ctf19**Δ* cells. *P*-values were obtained using Fisher’s exact test (n.s., nonsignificant ≥0.05, *P < 0.05; **P < 0.0001).

Finally, we aimed to address whether the observations made using our ectopic targeting system also held true at native pericentromeres. We analyzed CO frequency using a live cell reporter assay to measure recombination frequency in the vicinity of *CEN8*, as described earlier (Vincenten *et al.* 2015) in a *ctf19**-9A* mutant background. Indeed, as expected from our dCas9-based analysis, *ctf19**-9A* triggered a increase in CO frequency at *CEN8* (Figure 7A). CO frequencies in *ctf19**-9A* cells were increased less than what was observed in *ctf19**Δ*, which we speculate can be explained by a maintenance of DSB-suppression in this mutant, as opposed to what is seen in *ctf19**Δ* cells (Vincenten *et al.* 2015).

Together, these experiments, together with earlier work that linked Scc2/Scc4 function to local CO control (Vincenten *et al.* 2015), demonstrate that, also at native kinetochores, the NH_2_ terminus of Ctf19 is central to regulation of local CO repair of meiotic DSBs.

## Discussion

Control of DSB formation and meiotic CO repair is crucial for execution of the meiotic program. Too few, or too many, COs, COs placed at the wrong location, or DSB formation within at-risk regions jeopardize genome stability (Sasaki *et al.* 2010). Many factors influence CO formation, either by influencing DSB activity or post-DSB repair decisions (Keeney 2001; Hunter 2015), and manipulating these factors leads to global DSB and/or recombination effects. In addition, localized systems that control recombination within specific genomic regions exist (*e.g.*, Ellermeier *et al.* 2010; Vader *et al.* 2011; Vincenten *et al.* 2015; Nambiar and Smith 2018). One such localized mechanism is kinetochore-derived, and minimizes DSB activity and CO formation within pericentromeres (Vincenten *et al.* 2015). Here, we shed light on this mechanism. We developed a dCas9-based system to target individual kinetochore/Ctf19c subunits, and to dissect the mechanism of kinetochore-driven CO regulation. Using this system, we identified the Ctf19 protein as a nexus in mediating kinetochore-derived CO suppression.

Ctf19 is an RWD-domain containing protein, whose structural role within the kinetochore is linked to its assembly into the COMA complex (Schmitzberger and Harrison 2012; Schmitzberger *et al.* 2017). In addition, the unstructured NH_2_-terminal extension (amino acids 1–30) of Ctf19 functions as a phospho-dependent recruiter for the Scc2/Scc4 cohesin loader and activator complex (Fernius and Marston 2009; Hinshaw *et al.* 2015, 2017). We provide evidence that the contribution of Ctf19 to local CO regulation is mediated by this function: (i) abolishing the DDK-driven phosphorylation [by mutating 9 phosphoacceptor sites (ctf19-9A)] prevents CO suppression in a dCas9-targeted Ctf19 fusion, (ii) the NH_2_-terminal 30 amino acids (ctf19_1–30_) are sufficient for ectopic suppression, and suppression depends on the same phosphoacceptor sites, (iii) cotargeting Dbf4 (*i.e.*, DDK) with this NH_2_-terminal fragment strengthens CO suppression, in a manner that depends on the presence of phosphorylatable residues within Ctf19_1–30_, and (iv) mutating 9 DDK phospho-sites in Ctf19 (*i.e.*, ctf19-9a) leads to increased CO recombination at a native pericentromere. Taken together, our findings suggest that the NH_2_ region of Ctf19, through the recruitment of DDK-driven Scc2/4, impacts CO regulation. How does this pathway suppress CO formation? Local Scc2/4 function can alter cohesin function, by enhancement of chromosomal loading and via stimulation of cohesin’s ATPase activity (and likely also cohesin-dependent loop extrusion activity) (Petela *et al.* 2018) (Fernius and Marston 2009; Hinshaw *et al.* 2015, 2017; Davidson *et al.* 2019; Gutierrez-Escribano *et al.* 2019; Paldi *et al.* 2020). We proposed earlier that this alteration in cohesin function leads to a local shift in repair choice from interhomolog- into intersister-based repair (Kim *et al.* 2010; Vincenten *et al.* 2015). As such, local DSB repair will favor the eventual repair by using sequences present on sister chromatids. Intersister-based repair does not lead to CO formation (and interhomolog connections), and has been proposed to occur preferentially within pericentromeric regions (Vincenten *et al.* 2015). Our data strengthen the idea that a role of the kinetochore (and Ctf19) in minimizing meiotic COs revolves around its influence on cohesin function (Kuhl and Vader 2019).

CO suppression observed upon targeting of Ctf19 was modest in comparison to the CO suppression normally seen around native kinetochores; *e.g.*, compare the data in Figures 2–5 to those in Figure 7; also see Vincenten *et al.* (2015). We envision several possible (technical and biological) explanations for this discrepancy, and we addressed some of these in this study.

First, as we show in Figure 6, targeting of Ctf19 was not associated with local DSB suppression. At native kinetochores the Ctf19c suppresses DSB activity ∼fivefold within the 6 kb genomic regions that surround centromeres (Vincenten *et al.* 2015). A lack of DSB suppression in the case of ectopic Ctf19-targeting (as observed here) could explain (in part) why CO suppression is not as strong as what is seen around kinetochores. In agreement with this interpretation (and with our results upon targeting Ctf19 and its NH_2_-terminal fragments), interfering with cohesin function (via the *scc4**-m35* allele; Hinshaw *et al.* 2015) did not impair kinetochore-driven DSB suppression (Vincenten *et al.* 2015). These findings hint that DSB suppression at native kinetochores is related to the structural assembly of the Ctf19c/kinetochore.

Second, targeting of Ctf19 using our dCas9-system likely fails to reconstitute particular aspects of kinetochore organization. In fact, we initially set out to achieve exactly this, since such a condition would allow for dissection of functionalities. Kinetochore stoichiometry [each thought to contain two Ctf19c assemblies (Hinshaw and Harrison 2019; Yan *et al.* 2019)] is not recapitulated in single sgRNA-based targeting, which might explain lower suppression strength. Indeed, engineering a dCas9-molecule with two Ctf19 NH_2_ moieties enhanced suppression strength (Figure 5, C and D), suggesting that stoichiometry of kinetochore factors is important for CO regulation. In addition, certain aspects encoded in non-Ctf19 subunits of the kinetochore might collaborate with the “Ctf19-pathway” in mediating CO suppression. For example, DDK is recruited to kinetochores via Ctf3, and kinetochore-association of DDK is required for efficient phosphorylation of Ctf19 (Hinshaw *et al.* 2017). This aspect of kinetochore function is likely not recapitulated in Ctf19-targeted situations. Fusion of Dbf4 to Ctf19-dCas9 increased CO suppression, potentially caused by more efficient phosphorylation of Ctf19 (Figure 5, H and I). Furthermore, recent work has demonstrated that pericentromeres adopt a specialized 3D conformation, coordinately driven by local gene organization and kinetochores (Paldi *et al.* 2020). Three-dimensional organization might influence CO regulation, and it is conceivable that the ectopic sites studied here do not exhibit optimal gene organization to allow efficient formation of such a chromosome architecture.

Third, we do not know the efficiency and variability of dCas9-mediated targeting in individual cells: a subpopulation of cells might fail to recruit dCas9-fusion constructs, resulting in less efficient suppression frequencies.

Methods that allow for targeting of components of regulatory systems to ectopic sites (in isolation from binding partners or complexes) are useful tools to interrogate and dissect functional contributions (for example, see Kiermaier *et al.* 2009; Lacefield *et al.* 2009; Gascoigne *et al.* 2011; Ho *et al.* 2014). To our knowledge, we are the first to use dCas9-technology to establish such a method, and use this approach to manipulate CO formation via the recruitment of defined factors. Our method should be adaptable to allow the investigation and manipulation of other aspects of chromosome biology. Modulating CO frequencies is an engineering goal in crop development (Choi 2017; Lambing *et al.* 2017). Our approach could provide a basis to explore manipulation of recombination in plant breeding while eliminating the need for mutation of the genetic region of interest. Finally, combining our current system with the expanding repertoire of Cas9-versions and mutants (Knott and Doudna 2018) should facilitate multiplex targeting and inquiry of complex phenotypic behaviors. For example, in the case of the specific phenotype we studied here, targeting multiple kinetochore/Ctf19c subunits to adjacent loci should allow for more complete reconstitution and interrogation of kinetochore-driven regulation of DSB suppression and CO repair control.
